# Perceptions of specialists in the public sector, on the role and value of family medicine

**DOI:** 10.4102/safp.v64i1.5628

**Published:** 2022-11-09

**Authors:** Rihangwele Mukhinindi, Andrew J. Ross

**Affiliations:** 1 Department of Family Medicine, College of Health Sciences, University of KwaZulu-Natal, Durban, South Africa

**Keywords:** Family medicine, family physician, family specialist, specialist, perception, discipline, specialist discipline

## Abstract

**Background:**

Family medicine (FM) is often perceived to be a ‘lesser’ speciality compared with other disciplines, despite its importance as a generalist discipline in the healthcare system. Family physicians (FPs) provide comprehensive care at the district level and act as a gatekeeper for patient’s upward referral to other specialists. This study aimed to explore the perceptions of healthcare specialists other than FPs involved in registrar training regarding FM at the University of KwaZulu-Natal, South Africa (SA).

**Methods:**

This was a qualitative study, with seven consultants, other than FPs, who worked at three public sector regional hospitals using purposive sampling. Individual semistructured interviews were conducted, audio-recorded and transcribed verbatim and analysed thematically.

**Results:**

Four themes emerged (perception of FM as a medical speciality, role of FPs in the healthcare system and proposed National Health Insurance, FM registrars rotating in units and the scope of their training and how to improve the perceptions of FM by other specialities). Family medicine was regarded as a major and relevant specialist field with a significant contribution to make in advancing patients’ care. The country’s healthcare system is yet to make the best use of the FM specialist’s role in improving quality of care.

**Conclusion:**

The perceptions of FM from other specialists were generally positive and reinforced its importance in facilitating improved healthcare in SA. These specialists have high regard for FM and emphasised the large responsibility of practitioners.

## Introduction

International experience suggests that a well-trained medical generalist improves the quality of healthcare and reduces overall health costs.^1^ A review of the United States (US) literature showed that an increase in the number of primary care physicians in the health sector was associated with improved health outcomes, including all cases of cancer, heart disease, stroke, infant mortality, low birth weight, life expectancy and self-rated health. Starfield concluded that considerable health gains could be achieved by creating incentives to train more physicians in primary care.^2^ Studies in the US also showed that family physicians (FPs), as generalist physicians, contribute to improved patient care.^1,3^

The World Health Organization (WHO) reports that ‘primary health care (PHC) – now more than ever’ has identified excessive specialisation of healthcare providers and the narrow focus of many disease programmes as contributing to the fragmentation of healthcare services and poor health outcomes.^4,5^ The report observed that a better balance between specialised curative care, first contact care and health promotion has contributed to significant improvements in health outcomes.^4^

In 2009, at the 62nd World Health Assembly, a resolution on PHC (including health system strengthening) recommended, among other things, the need to train and retain adequate numbers of health workers with an appropriate skills mix, including primary healthcare nurses, midwives, allied health professionals and FPs, in order to ensure universal access to healthcare.^6^

It was only in late in 2007, after much lobbying, that the Health Professions Council of South Africa (HPCSA) approved a new speciality called family medicine (FM), with the specialist generalist referred to as a FP. The FP is considered to be an expert, or specialist generalist, who has a master’s degree and provides whole-person care by taking care of the physical, mental, social and spiritual health, with consideration of the patient’s fears and concerns about their health issues.^7,8^ Family medicine integrates several medical specialities, being concerned with the holistic approach to patient care in which the individual is seen in totality and in the context of their family and community. In addition, the FP continues and coordinates the care when referral to multiple specialists is needed.^7^ They also play a key role in providing comprehensive care at the district level, being an important generalist discipline that provides care and acts as a gatekeeper for upward referral of patients to specialists.^9,10,11^

Following the recognition of FM as a speciality, each of the eight departments of FM in South Africa (SA) committed themselves to training 12–15 FPs per year, with the first cohort of graduates having become available for deployment in 2011.^9,12^ The Green Paper on National Health Insurance (NHI),^13^ the draft document on re-engineering PHC^14,15^ and the Government Gazette of 12 August 2011 all speak of the important role of the specialist FP in healthcare delivery in SA. The FP, as a multiskilled generalist, is seen by the National Department of Health (NDOH) as a key member of the team that will help deliver on priority health outcomes.

In addition, in 2019 the HPCSA, after acknowledgement and recognition of the role of FM, changed the internship requirements for newly qualified medical doctors to a compulsory 6 months rotation in FM while reducing the other speciality rotations, re-emphasising its important role.^5,16^ However, despite the NDOH’s obvious commitment to FM through the Green Paper, the re-engineering of PHC, the additional time allocated to FM in the internship programme, as well as the evidence that supports the effectiveness of well-trained generalist, it has been an ongoing struggle to convince the provincial DOHs to commit resources to the creation of FP posts. This was highlighted in the human resources for health 2030 document, which estimated that the country was short of 888 FPs.^17^ In addition, those FPs who go into private practice after their four years of training are not remunerated at the same rates as other specialists by most medical aid companies in SA, which creates the impression that they are not regarded as ‘real’ specialists.^18^

Although there is considerable literature on why students do not choose to specialise in FM,^19^ and on what FPs think of their career choices, little local literature is available on the perceptions of other specialists on the role of FM in SA and whether or not they perceive it as an attractive specialisation.^20^ The aim of this study was therefore to assess the perceptions of other specialists working in the public sector on the role and value of FM. It is hoped that this research will improve the understanding of and working relationships between FM and other specialists, which are often strained. It is important to understand why the role and place of FM still remains unclear to other specialists, despite the presence of FM training in the country since 2007.

## Research design and methods

This was an exploratory study using semistructured one-to-one interviews to collect qualitative data with seven participants who were purposefully selected. As specialists play a prominent role in the training of doctors in SA, and often set the tone of how colleagues working in other domains are perceived, the researcher purposefully selected heads of clinical units (HCUs) and senior specialists in the seven domains through which interns rotate (internal medicine, anaesthesia, general surgery, psychiatry, obstetrics and gynaecology, orthopaedics and paediatrics; FM departments, the eighth, were not included). In addition, as the FM registrars rotate through these domains during their 4-year training, the assumption was that the HCUs would be familiar with FM and FM training.

The study was conducted in the three regional teaching hospitals in Durban (King Edward VIII Hospital [KEH], Addington Hospital [ADH] and Mahatma Gandhi Hospital [MHGH]) that are linked to the University of KwaZulu-Natal (UKZN) as part of the FM registrar training programme. The HCUs and senior specialists who participated in the study supervised FM registrars during their 3–4 months’ rotation in that domain in their second or third year of training. Five of the seven interviews were conducted in person in the specialist’s office, while two were conducted virtually via Zoom (Zoom Video Communications, San Jose, California, United States) to accommodate those who requested a Zoom rather than an in-person interview.

A semistructured interview (SSI) guide, based on information from the literature, was used to ensure that important topics were covered in the interviews and enabled the specialists to air their views, opinions and perceptions towards FM as a discipline. The SSI was developed by the author with the assistance of the co-author. The SSI covered the following:

- What is your perception of FM as a medical speciality?
- What do perceive as the role of the FPs in the healthcare system and proposed NHI?
- What are your perceptions of FM registrars rotating in your unit and the scope of their training?
- What do you think would improve the perceptions of FM by other specialities?

All interviews were audio-recorded, transcribed verbatim and analysed using a deductive approach aided by NVivo 12 Pro software (QSR International, Doncaster, Australia).^21^ The following steps were used during the analysis: (1) familiarisation, (2) development of a coding index, (3) indexing, (4) charting, (5) mapping and (6) interpretation.

Participants were allocated alphabetical letters in place of their names to ensure anonymity, with confidentiality maintained by omitting their personal information in the transcripts.

To address trustworthiness, the data analysis was carried out by the researcher through the construction of a coding index and interpretation of the findings. The analysis followed a clear stepwise process that could be audited with the help of NVivo, which helped to guard against data loss and ensured trustworthiness, credibility and quality.

### Ethical considerations

Ethical clearance was obtained from the Biomedical Research Ethics Committee of UKZN (ref. no. BREC/0002632/2021). Permission to conduct the research was given by the KwaZulu-Natal Department of Health Research Committee (ref. no. KZ 202105/019) and gatekeeping permissions were obtained from all the three hospitals involved in the study: King Edward VIII Hospital (ref. no. 2/7/1/06/2021), Addington Hospital (ref. no. 9/2/3/R) and Mahatma Gandhi Hospital (ref. no. 14/2020). Informed consent was obtained from all participants before the interviews.

## Results

Seven specialists participated in the study, these being from the disciplines of orthopaedic surgery, internal medicine, general surgery, paediatrics, psychiatry, obstetrics and gynaecology and anaesthesiology. The range of work experience was 3–24 years, with four being HCUs and three being senior specialists, who were interviewed if the HCU was not available.

### Theme 1: Perceptions about family medicine

The participants had a range of perceptions about FM, such as general practice, family health practice, general practice in the community, district practice, primary healthcare, medical emergencies team work and holistic care. Some of these perceptions were based on their experiences growing up where the ‘general practitioners were our first call’ (Participant A). Others were influenced by meeting pioneers of FM during their undergraduate training, including Prof. Bruce Sparks (previous HOD of FM at the University of the Witwatersrand), who was regarded as:

‘[O]ne of the pioneers of family medicine as a separate discipline on the Wits circuit. A fantastic guy, who put family medicine on the map. I think Family Medicine is a very real specialty that requires a lot of skills, just as emergency medicine is a specialty, I think family medicine is very critical.’ (Participant H, 50–60 years old, male)

A few participants did not have any experience working with FP outside the formal training environment, as FM was not part of the internship rotations during their internship and community service. However, there were a number of participants who had very positive experiences of working with FPs during their internship or community service:

‘As an intern I did a rotation in family medicine where I spent a lot of time in out-patient department and I enjoyed the emergency department, which was run by family medicine. I learned a lot about resuscitations and how to manage medical emergencies.’ (Participant E, 40–50 years old, male)

Others had worked with FPs as junior doctors:

‘I remember a hospital where I worked as a junior doctor and witnessed the teamwork by FPs and the other members of the treatment team, including physiotherapists, social workers, psychologists and many others.’ (Participant G, 30–40 years old, male)

Others were aware of FPs who worked in:

‘Hospital emergency departments that don’t have dedicated emergency specialists, but this was not obvious because family physicians all over the country are managing these departments, and the patients that present there with different medical emergencies end up receiving holistic care, which prevents repeated hospital admissions.’ (Participant C, 50–60 years old, male)

Participants rated FM as harder than other disciplines to specialise in because of its breadth of scope of practice:

‘Any speciality is difficult, I think with family medicine it is particularly difficult because it also requires the clinician to be quite broad based in terms of their knowledge. If you look at one of the disciplines like surgery it is very myopic whereas you find family medicine tends to be broad-based.’ (Participant C, 50–60 years old, male)

Others agreed that:

‘A family physician needs to know everything, about everything. Family medicine tends to be broad-based, it requires knowledge of other specialties.’ (Participant F, 50–60 years old, female)

Participants felt that there was a need for more organised legislation to differentiate general practitioners (GPs) from FPs, especially for the nonmedical public, many of whom assume them to be the same. Most GPs call themselves ‘family doctors’, with patients tending to regard them as FM specialists. The reason why this differentiation is important is because of the poor standard of care provided by some GPs, which creates a negative perception of all generalist doctors, including FPs:

‘I think family physicians, or whoever is responsible, needs to take this seriously. The confusion is very clear, and most of the time it’s associated with bad medical practice and will definitely drag the FM specialist down. Patients end up looking for “real specialists” after that perceived poor treatment.’ (Participant C, 50–60 years old, male)

‘A general practitioner [*GP*] is not a specialist, a family physician must have more knowledge than a general practitioner, as GP may not have that the vast knowledge that is expected of a FP.’ (Participant F, 50–60 years old, female)

### Theme 2: Role of the family physician in the healthcare system and National Health Insurance

This theme consisted of two subthemes, the first relating to the role of FPs in the healthcare system in general and the second to the proposed NHI scheme.

#### Subtheme 1: Role of family physicians in the healthcare system

Participants identified three areas where they felt that a FP should work, which included community health centres (CHCs), district-level care and regional hospitals. Some felt that for effective healthcare, FPs are needed at all levels of care. However, the majority of specialists were of the opinion that the district level should be given priority:

‘I think family physicians should be at a district level of care. The reason is that’s the first port of call for most patients and then from there a referral to other specialties. We find that patients often come to specialties either being inappropriately referred, or undermanaged or inappropriately managed. So, if a family physician is at that space, they’re qualified enough to make decisions and manage patients before they warrant referral to other specialties.’ (Participant A, 20–25 years old, male)

The specialist colleagues saw the role of the FP primarily as a skilled care provider who was involved in managing patients; preventing complications; detecting, managing and referring complicated patients to higher levels of care; providing holistic and evidence-based care; and acting as a bridge between district hospitals (DHs) and specialists. They also saw the FP as someone who was able to fill the gaps in the DHs, provide supervision and training to medical officers or interns working under them, improve communication among medical practitioners, provide leadership and management skills at the DH and coordinate multidisciplinary and interdisciplinary care.

One of the participants said:

‘FM is a good discipline to have, as it is able to investigate and manage the various conditions.’ (Participant F, 50–60 years old, female)

‘Family medicine provides the most effective treatment for patients, as, most of the time they relying on history and examination only with very limited investigations. I think that there is no discipline more clinical than that.’ (Participant D, 50–60 years old, male)

‘It’s critical to have family medicine specialist as managers of hospitals, because most times if you have other specialities as managers, their vision gets tunnelled to the needs of that discipline, whereas family physicians find it easy to form a multidisciplinary team and also work as part of the interdisciplinary team.’ (Participant C, 50–60 years old, male)

#### Subtheme 2: Family medicine in the National Health Insurance scheme

There was little clarity about how the NHI will work, with one participant stating: ‘To be honest, the NHI is something that I’m not exactly clear on as to how the government is going to roll it out’ (Participant C). Despite this uncertainty, a participant felt that ‘family medicine will be a pivotal pillar’ (Participant C) with a key role and FPs acting as gatekeepers to higher services and ensuring cost-effective, high-quality care at an appropriate level:

‘The NHI is obviously going to provide care for the entire population of people and family medicine will obviously be those care gate keepers of the service. If you’re going to look at health in the future, one has to be cost-conscious, one has to be quite specific as to how one deals with patients and it will position the family medicine specialist as a gatekeeper. You’ll find unnecessary referrals get put away, you’ll find this better cost effectiveness, better care in place. I think family physicians should be at the forefront of the NHI in terms of trying to manage that system appropriately.’ (Participant A, 20–25 years old, male)

‘The main aim of NHI is to improve access to quality health services for all South Africans, irrespective of whether they are able to afford it or not. I truly don’t think this will be possible without family physicians, they are the only discipline which can provide holistic care.’ (Participant D, 50–60 years old, male)

### Theme 3: What are your perceptions of family medicine registrars rotating in your unit and the scope of their training?

The FM registrars rotate through all the major domains for 3–4 months in their second or third year. They have specific learning outcomes that they need to achieve, this being facilitated by a learning plan that is drawn up at the beginning of the rotation in consultation with the HCU. This theme was further divided into two subthemes: (1) having FM registrars in their unit and (2) scope of training.

#### Having family medicine registrars in their unit

The respondents made a number of comments about having the FM registrars rotating in their units. They observed that while they ‘leant from them’, it was also ‘a learning curve’, with most being regarded as being ‘open to learning’, as they ‘seemed to love rotating in specialist blocks’. They were regarded as being ‘a positive influence in the department’, with some needing help ‘with referring appropriately’ to ensure that patients were managed correctly.

One of the participants said:

‘It’s been a learning curve for me as it is relatively new to me to deal with FM registrars.’ (Participant C, 50–60 years old, male)

‘I think all registrars from all specialties should go through a family medicine rotation to allow them to learn how to refer appropriately and how to communicate with people on different levels of care.’ (Participant E, 40–50 years old, female)

Three different opinions emerged about the training of FM registrars, with some contending that there was adequate training in that speciality to enable them to function as generalist at a district level. Others were of the opinion that the duration of the rotations was too short, and to consolidate their training, FM registrars should strive to continue to practise at the same level as they did when working in that department, despite now rotating in another department:

‘Just when the family medicine registrar gains the knowledge needed to effectively manage the cases we deal with in the department, you find that their rotation comes to an end and they move to something totally different.’ (Participant A, 50–60 years old, male)

‘We should think about how to maintain relationships with the domains after the rotation ends and should have some form of continued social interaction to maintain those formed during the FM registrar rotation.’ (Participant D, 50–60 years old, male)

### Theme 4: Improving the perceptions of family medicine by other specialities

The participants expressed five ways that they thought would improve how FM was perceived by other disciples, these included: better recognition by the Provincial and NDOH; improved remuneration; more effective legislation; specialists post creation and for sustainability of the posts with necessary support. Participants observed that:

‘[T]here is an urgent need to make FM more recognizable, because if you are not in a teaching hospital you easily forget what FM is. I mean, if I was not interacting with FM registrars on a regular basis I’m not sure if I would remember, it’s not like I regularly have to refer a patient to them.’ (Participant F, 50–60 years old, female)

‘The unclear differentiation with GPs doesn’t help matters, because if you are in private healthcare, for example, it’s not very clear what FM does that cannot be performed by GPs for the patients. This makes the referral system a challenge, but in public it’s better, because we are not chasing patients as much.’ (Participant H, 30–40 years old, male)

‘The lack of posts after completing FM in the country is a serious challenge. If one is going to finish and only get employed as a medical officer, whereas other departments, like internal medicine and paediatrics, have so many posts, I don’t see how people would value the discipline the way they value other disciplines.’ (Participant G, 30–40 years old, male)

## Discussion

### Theme 1: Perception of family medicine as a medical speciality

Participants in this study were positive in their perceptions towards FM. They found that the FP, who they saw as a specialist generalist working at a DH or in the community, was an integral part of the delivering services at that level. It was encouraging that specialist colleagues saw the FP as more than a GP but as a specialist able to provide broader, more comprehensive, team-based and coordinated care.

The South African Academy of Family Physicians (SAAFPs), the professional body of FP in SA, recently released a recent positional article, which identified a DH, a CHC or in the community as the best place for a FP to work at. In addition, the SAAFP has motivated to the National and Provincial Departments of Health for FP posts to be created at DHs or CHCs.^22^

### Theme 2: The role of family physicians in the healthcare system and National Health Insurance

**FIGURE 1:**
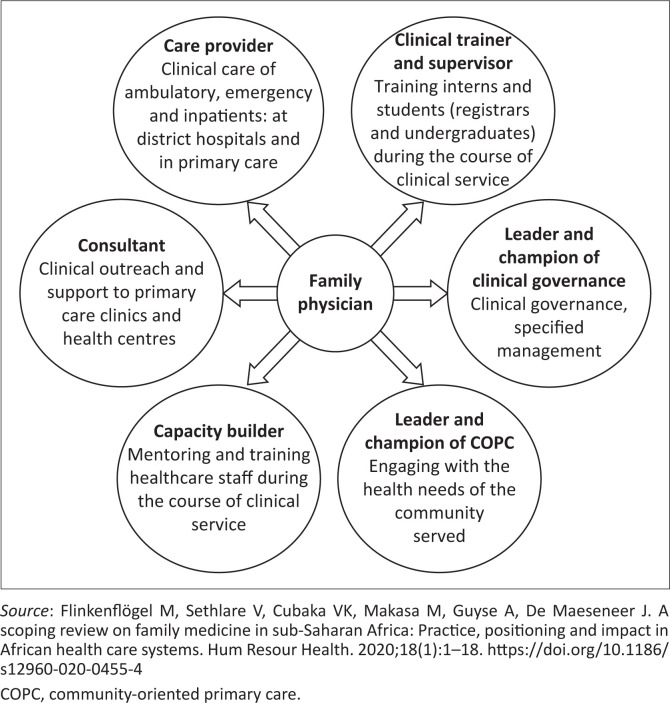
Roles and competencies expected of a family physician in South Africa.

As a result of their personal experiences and involvement in the registrar training programme, the specialists who participated in this study were able to identify several of the key roles of a FP. They identified quality of clinical care and a link to specialist services as a core responsibility of the FPs. However, SAAFP has identified five additional roles of a FP, which include consultant, capacity builder, supervisor, leader of clinical governance and champion of community-oriented primary care (COPC) (see Figure 1).^23,24,25^

It is not surprising that specialists in teaching hospitals were unaware of the some of the wider roles of the FP (such as COPC), as they are predominantly concerned with ensuring that registrars in FM have sufficient clinical expertise to manage patients in that domain, with the focus of vertical specialist care on specific patients in that speciality and not the community. The FM registrars in departments would have been expected to be involved in activities related to clinical governance (audits – perinatal, morbidity and mortality) and teaching. These may not have been recognised by those who participated in the research, as these activities are part of the day-to-day work in those departments, and FM registrars would have been expected to participate in activities such as audits of Caesarean section meetings, teaching junior doctors minimum safety standards for safe Caesarean sections, conducting a maternal death review, conducting a perinatal morbidity and mortality meeting and the perinatal problem identification programme, to name just a few.

Healthcare professionals often opt out of leadership roles, leaving others to make important decisions about how the services should be run.^26,27^ This important role was recognised by those who participated in the study. There is a need to reinforce leadership training, and careful thought needs to be given as to how it could be strengthened in the registrar training, a key role of the FP is to participate in clinical governance activities within the district health system.^28^

### Theme 3: Perceptions of family medicine registrars rotating in the units and the scope of their training

With regard to the training of FPs, specialists had very positive views on FM registrars who rotated through the disciplines. It is encouraging that learning was mutual, FM registrars had a positive influence on the departments that they rotated through and FM registrars were keen to learn. In accordance with the unit standards that guide the training of FPs in SA, registrars are expected to learn to evaluate and manage patients with both undifferentiated and more specific problems cost effectively according to the biopsychosocial approach. Learning in each domain is guided by a learning plan, which helps the registrar to focus on those aspects that he or she needs to master during the rotation in each domain.^29,30^

If FPs are to fulfil the roles suggested by the specialists, to staff DHs and be the link between generalist and specialist services, then there is an urgent need to upscale the training of FPs and create FP posts at DHs and CHCs, as suggested by the participants in this study.

The SAAFP has suggested a short-term goal of one FP at every DH as well as every CHC or subdistrict health.^1^ Currently, if all new graduates entered the public sector, it would take 18 years to fill this gap. However, in the current economic climate where posts are being frozen and few FPs get specialist’s posts after completion, this may be a dream that may never be reachable.^18^

It is not surprising that there was confusion among the specialists about the NHI and what it might look like, as the NDOH has not provided details on what the NHI will eventually look like. However, it was encouraging that the vertical specialists recognised the important role the FPs as specialist generalists could play in the NHI. The NHI has the potential to be a game changer in SA if performed well, with FM as the core discipline in ensuring comprehensive, affordable, quality health for all in SA.

Although the medical community is still unsure of how the NHI will be implemented, it is clear that from international experience, that there has not been a model anywhere that has worked without involvement of people working in communities or at the primary care level.^31^ Family medicine is aligned with the ideals of the NHI, which are to provide quality primary care in a person-centred manner which ensures access, continuity and coordination of care for all patients.^32,33,34^

### Theme 4: Improving the perceptions of family medicine by other specialities

A 2017 study on the influence of FP district health system performance in SA^35,36^ failed to show a measurable effect on health outcomes at facilities where FPs were based. This was thought to be because of the low number of FPs working in the public sector across SA. Despite international and local evidence and support from regional specialists of the value of FPs,^36,37^ provincial departments of health have not created FP posts at most DHs or CHC.

Until there is sufficient local evidence to persuade the local health authorities of the importance of FM or a significant shift in the policy and practices which determine how posts are allocated and funded, FM will continue to be a Cinderella discipline, forever failing to reach its potential to contribute to the provision of comprehensive quality care in SA.

There are a number of initiatives currently underway to address some of the issues identified. These include participation in a policy review on the role of DHs (which includes appropriate staffing), a number of research projects to document the impact that FM and FPs can have on service delivery as well as lobbying for medical aids to compensate FPs as specialists. It is hoped that these initiatives will influence policymakers.

## Conclusion and recommendation

With SA moving towards the NHI, there is a need for clear delineation of roles and values of the FP in order to improve teamwork among different professionals within the healthcare services for the requisite operational efficiency. The potential exists for the burden of disease to be eased with greater involvement of FM in PHC. However, the shortage of specialist posts for FM registrars upon completion of the training has been highlighted by participants. Unless addressed, these shortages will negatively affect the potential impact which FM could have on patient care.

### Limitations

There are inherent limitations with qualitative studies because of the small number and purposeful selection of participants. In addition, this study was only conducted in one province of SA and is limited to the views of specialists at one university of the country; therefore, the results of this study may not be generalised to other public healthcare institutions in the country.
